# Novel High-Strength and High-Temperature Resistant Composite Material for In-Space Optical Mining Applications: Modeling, Design, and Simulation at the Polymer and Atomic/Molecular Levels

**DOI:** 10.3390/ma17194723

**Published:** 2024-09-26

**Authors:** Hadarou Sare, Dongmei Dong

**Affiliations:** Department of Physics and Astronomy, Rowan University, Glassboro, NJ 08028, USA; sareha36@rowan.edu

**Keywords:** polymers, electrochromic, photochromic, modeling, composite material, structure, asteroid mining

## Abstract

This study explores the modeling, design, simulation, and testing of a new composite material designed for high-strength and high-temperature resistance in in-space optical mining, examining its properties at both the polymer and atomic/molecular levels. At the polymer level, the investigation includes mechanical and thermal performance analyses using COMSOL Multiphysics 6.1, employing layerwise theory, equivalent single layer (ESL) theory, and a multiple-model approach for mechanical modeling, alongside virtual thermal experiments simulating laser heating. Experimentally, porous Polyaniline (PANI) films are fabricated via electrochemical polymerization, with variations in voltage and deposition time, to study their morphology, optical performance, and electrochemical behavior. At the atomic and molecular levels, this study involves modeling the composite material, composed of Nomex, Kevlar, and Spirooxazine-Doped PANI, and simulating its behavior. The significance of this work lies in developing a novel composite material for in-space optical mining, integrating it into optical mining systems, and introducing innovative thermal management solutions, which contribute to future space exploration by improving resource efficiency and sustainability, while also enhancing the understanding of PANI film properties for in-space applications.

## 1. Introduction

Because of the valuable assets that asteroids possess, the examination of asteroids for forthcoming excavation intentions has garnered significant focus [1,2,3,4]. For these operations, one must contemplate the following: (i) the spacecraft rendezvous and navigation phase, during which precise management of the spacecraft’s dynamics is necessary while the spacecraft traverses the proximity of the asteroid being apprehended, (ii) the asteroid apprehension phase, and (iii) the optical excavation phase [1].

Although rendezvous assignment and docking [1], spacecraft rendezvous and close-range maneuvering [1,5,6], and spacecraft direction in rendezvous assignments [1,7] have been extensively explored in the literature, asteroid excavation without digging (optical excavation) has not been extensively examined. To tackle this void, the primary aim of this document is to model, design, simulate, and test experimentally a high-strength and high-temperature resistant composite structure material for in-space optical excavation (asteroid excavation). In other words, this paper consists of (i) modeling and designing a composite material at the polymer and atomic/molecular levels, (ii) investigating the mechanical performance (high strength) of the composite material at the polymer and atomic/molecular levels for in-space optical mining, (iii) investigating thermal performance (high-temperature resistant) at the polymer and atomic/molecular levels of the composite material for in-space optical mining, and iv) experimental testing of the composite material.

The objective of the mechanical performance investigation of the material designed and modeled at the polymer level is to figure out the best combinations of materials suitable for use in space asteroid mining (optical mining). For this paper, we only show the modeling and simulations of a composite structure material made up of Nomex (outer layer), Kevlar (supporting layer), and Spirooxazine-Doped PANI film (core layer).

It is important to mention that multiple combinations made up of the following were modeled and simulated in COMSOL: Nomex, Kevlar, Aerogel, carbon epoxy, Glass vinylester, Kapton, polyvinyl chloride (PVC) foam, Polyethylene Terephthalate (PET), Polyamide (Nylon), Polyimide, Aluminized mylar, PTFE (Teflon), Polypropylene, Silica aerogel, high-temperature fabrics, carbon/carbon composite. Based on the listed materials, the combination of Nomex/Kevlar/Spirooxazine-Doped PANI film stands out as the best combination to be considered as a high-strength composite structure material for in-space optical mining.

The objective of the thermal investigation is to determine if the selected composite material (material made up of Nomex, Kevlar, and Spirooxazine-Doped PANI film) based on the mechanical performance investigation is suitable for optical mining (asteroid mining).

To examine the thermal efficiency of the composite material designed and modeled at the polymer level, a simulated trial was established using COMSOL. Within this setup, a laser heats a composite consisting of Nomex, Kevlar, and Spirooxazine-Doped PANI film. The laser moves both inward and outward radially as time progresses, while the composite material rotates on its platform [8]. Initially, for the examination of the mechanical aspects of the composite material modeled and designed at the polymer level, the analysis contrasted the following frequently utilized techniques [9]: (i) an equivalent solitary layer principle [8], employing first-order shear deformation theory (ESLFSDT) [8], (ii) layerwise elasticity theory [8], and (iii) a multiple-model approach [8]. It is noteworthy that these identical methodologies were applied and fully described in detail in our previously published article [8].

Next, for the thermal analysis of the composite material modeled and designed at the polymer level, the composite material fabricated and simulated mechanically (mechanical characteristics simulations) underwent heating from a laser that traverses radially inward and outward over time, while the composite itself rotates on its platform [8]. The transient thermal reaction of the composite was acquired by modeling the incoming heat flux from the laser as a spatially distributed heat source on the surface [8]. The mean, peak, and nadir temperatures, alongside the utmost temperature disparity across the composite, were recorded at each computational increment [8]. The temperature dispersion throughout the entire composite material was documented at specified intervals of output time. It is noteworthy that this identical approach was employed in our previously published manuscript [8].

Fourthly, for the experimental investigation of the composite material modeled and designed at the polymer level, a thin PANI layer was successfully synthesized via electrochemical polymerization on a flexible substrate [10]. The quantitative analysis of colorimetric [10], optical [10], and structural evolution [10], alongside the EC properties of the electrodeposited PANI film [10], were meticulously examined across various potentials [10]. The Raman spectra delineate the underlying mechanisms driving the observed color alterations in response to external stimuli [10]. The synthesized film underwent scrutiny using a diverse array of characterization techniques [10], including scanning electron microscopy (SEM) [10], colorimetric analyses [10], UV-vis spectroscopy [10], Raman spectroscopy [10], and cyclic voltammetry [10]. It is pertinent to note that this identical methodology [10] was applied and fully described in detail in our previously published manuscript [10]. The contributions of these methodologies to understanding the following properties of the PANI film were described in detail in our published manuscripts [10]: surface morphology, electrochemical dynamics, and molecular structural evolution [10].

Following the initial design, modeling, and testing of the composite material at the polymer level, we advanced our approach by designing, modeling, and simulating a novel composite material composed of Nomex, Kevlar, and Spirooxazine-Doped Polyaniline (PANI) at the atomic and molecular levels. In this refined process, we meticulously considered constraints such as chemical structure, bonding, and physical interactions, including Van der Waals forces, to ensure an accurate representation of the material’s behavior.

As part of our design concept, the composite material was engineered with interwoven or layered Nomex and Kevlar polymer chains, creating a robust and flexible network. A thin film of Spirooxazine-Doped PANI was then strategically deposited onto this composite network, enhancing its functional properties through the incorporation of photochromic and conductive features. Subsequent simulations of the composite material were conducted, and the results were rigorously compared to those obtained from earlier modeling and simulations performed at the polymer level. This comparative analysis allowed us to validate the material’s performance and optimize its structural and functional characteristics for potential applications in advanced technological fields.

The uniqueness and contributions of this endeavor are delineated as follows: (i) pioneering material advancement for in-space optical mining application. This initiative introduces an original composite material endowed with outstanding high-strength and high-temperature resistance tailored explicitly for in-space optical mining. The development of this material may encompass groundbreaking amalgamations of polymers, ceramics, or alternative cutting-edge materials. (ii) Fusion of composite material with optical mining systems. This initiative showcases the formulation of simulation models that seamlessly integrate the composite material into the overarching optical mining framework. This entails deliberations on the material’s interaction with mining apparatuses, energy reservoirs, and other constituent elements to optimize the entire mining continuum. (iii) Thermal regulation solutions for the optical mining milieu. This endeavor unveils innovative remedies for thermal regulation in environments characterized by elevated temperatures encountered during optical mining. This encompasses simulations demonstrating the material’s adeptness in heat dissipation or resistance to thermal deterioration, thus ensuring the robustness and endurance of mining paraphernalia. (iv) Understanding material dynamics in the space environment. Noteworthy emphasis is placed on the distinctive challenges posed by the space milieu, such as microgravity and vacuum conditions. This initiative contributes to comprehending how the material reacts in these conditions and its ability to endure or adapt to the rigors of space mining. (v) Assessment of the material’s impact on resource efficiency and sustainability in space missions. This entails considerations of recycling, reusability, and decreased reliance on earth-derived resources for space expeditions. (vi) Fostering interdisciplinary collaboration. This endeavor fosters synergies among researchers from diverse domains, including materials science, aerospace engineering, optics, and mining engineering. This interdisciplinary synergy can furnish holistic insights into the challenges and prospects linked with in-space optical mining. (vii) Advancing future space exploration. This undertaking enriches the broader domain of space exploration by furnishing solutions for resource extraction and utilization in space, vital for sustainable and enduring human presence beyond Earth. (viii) This empirical exploration enhances our comprehension of the Spirooxazine-Doped PANI film structure and the electrochemical and correlated optical attributes, thereby offering deeper insight into dual-function EC charge storage polymers and other energy-related functional materials.

The outcomes of the mechanical and thermal explorations evince that the composite material comprising Nomex, Kevlar, and Spirooxazine-Doped PANI film manifests potential as a high-strength and high-temperature resistant composite structural material for in-space optical mining endeavors.

## 2. Model Development and Experimental Testing of the Composite Material at the Polymer Level: A Composite Material Made Up of Layers of Nomex, Kevlar, and Spirooxazine-Doped PANI Thin Film

### 2.1. Model Definition for the Mechanical Investigation

In this context, the authors of this paper perform an eigenfrequency analysis and a frequency-domain analysis on a composite structural material composed of the following three layers: Nomex (outer layer), Kevlar (reinforcement layer), and Spirooxazine-Doped PANI film (core layer). We utilize the following modeling strategies as described in [8]: (i) equivalent single layer theory, (ii) layerwise theory, and (iii) a multiple-model method. These approaches are combined in the through-thickness dimension of the composite material, integrating the ESL and layerwise theories.

*(1) Geometry and boundary conditions:* The composition and specifications of the composite material consisting of Nomex, Kevlar, and Spirooxazine-Doped PANI film are outlined in our previously published article. The boundary conditions and loading are detailed as follows [8]: (i) The fixed end of the composite structure is positioned at the shorter end. (ii) Pressure excitation is applied to the surface of the composite material as the load.

The equation and parameters governing the magnitude and constant amplitude of the force are detailed in our published article (refer to [8]).

*(2) Material Properties:* The composite material is composed of the following distinct materials: Nomex (outer layer), Kevlar (reinforcement layer), and Spirooxazine-Doped PANI film (core layer). Details and properties of Nomex and Kevlar can be found in our previously published article [8]. The focus of this paper is on the Spirooxazine-Doped PANI film, which constitutes the inner layer of the composite structure alongside Nomex and Kevlar.

The Spirooxazine-Doped PANI film, with a thickness of 0.75 mm, serves as the innermost material within the structure. The density of the material can be seen in Table 1. The orthotropic material characteristics are outlined in the table below [8,11,12,13,14,15,16,17].

Further details regarding the stacking sequences, material orientations, and layer thicknesses can be found in our published article [8].

*(3) Finite Element Mesh:* The composite material undergoes both in-plane and out-of-plane discretization. A triangular mesh, as illustrated in our previously published article [8], is utilized in the plane. The discretization along the material’s thickness direction is detailed in Table 2.

### 2.2. Model Definition for the Thermal Investigation

The thermal investigation of the composite material, as detailed in our previous publication [8], involves heating a 2-inch-thick composite comprising Nomex, Kevlar, and Spirooxazine-Doped PANI film using a 10 W laser for one minute. The laser oscillates while the material rotates on its stage. This material size corresponds to that modeled in the earlier mechanical investigation. The same methodologies from our previous work [8] are applied here.

Assuming effective thermal insulation, heat loss occurs only through radiation from the upper surface to the chamber walls, set at 20 °C. The laser’s thermal impact is simulated as it moves across the rotating material surface, utilizing COMSOL’s Moving Mesh–Rotating Domain feature and a Gaussian distribution of the laser’s heat load.

Temperature profiles across the material can be viewed from external or rotating perspectives. The surface emissivity of the composite material is approximately 0.8, with all laser heat deposited solely at the material surface because of the laser’s opaque wavelength.

Meshing employs a triangular swept mesh with a single thin element through the thickness, and the solver’s relative tolerance is slightly reduced to capture the effect of the moving heat load. While a finer mesh and tighter solver tolerances could enhance peak temperature predictions, average and minimum temperature predictions would remain largely unaffected.

### 2.3. Experimental Method to Fabricate and Test the Material

In this study, we focused on developing and testing a PANI thin film rather than a Spirooxazine-Doped PANI film because of the relative ease of fabricating PANI thin films in our laboratory setting. Both materials share similar electrochromic properties, making this a viable alternative for initial testing. The PANI thin film exhibits electrochromic behavior when voltage is applied, while Spirooxazine-Doped PANI demonstrates electrochromic properties in response to sunlight. This distinction allows for diverse applications where either electrical control or light sensitivity is desirable. By concentrating on PANI, we could efficiently conduct experiments and gather data, laying the groundwork for future exploration of Spirooxazine-Doped variants.

All-encompassing details concerning the materials, PANI electrodeposition, PANI characterization, and circuit design for the EC films are available in our previously published article (refer to [10]). This article offers an extensive analysis of these elements, providing invaluable insights for deeper comprehension and continued exploration within this domain.

## 3. Model Development of the Composite Material at the Atomic and Molecular Levels: Design and Development of a New Composite Material Made Up of Nomex, Kevlar, and Spirooxazine-Doped PANI

### 3.1. Considerations for the Development of the Composite Material

Given that the objective is to create a material for in-space optical mining applications, we aimed to develop a composite consisting of Nomex, Kevlar, and a photochromic material specifically designed for asteroid mining.

The photochromic material will replace the PANI thin film used in our previous work, as documented in our published article [8]. This material must be capable of changing color upon exposure to sunlight, enhancing the functionality of the composite.

Key considerations for this development include the following. (i) Integration: The photochromic material must be seamlessly integrated into the composite without compromising the mechanical properties of Kevlar and Nomex, maintaining their inherent strength and durability. (ii) Stability: The photochromic material must withstand the extreme conditions of space, including radiation, temperature fluctuations, and vacuum environments, to ensure long-term stability and performance. (iii) Reversibility: The photochromic change should be reversible, allowing for multiple cycles of color change in response to sunlight exposure, which is critical for the dynamic conditions of space. (iv) Performance: The selected photochromic material should enhance the composite’s suitability for in-space asteroid mining, leveraging the strengths of Nomex and Kevlar to provide a robust and versatile solution.

After extensive investigations and simulations, Spirooxazine-Doped PANI emerged as the optimal choice. This material is renowned for its ability to change color under sunlight exposure and its durability and stability in harsh environments.

By replacing the PANI thin film with Spirooxazine-Doped PANI, the resulting composite material—comprising Nomex/Kevlar reinforced with a Spirooxazine-Doped PANI thin film—will benefit from enhanced physical interactions and hydrogen bonding, defined as follows:

*(1) Physical Interaction:* The Nomex and Kevlar fibers will be interwoven to form a strong, three-dimensional network. The Spirooxazine-Doped PANI thin film will be deposited onto this network, potentially through methods like spin-coating or vapor deposition. The PANI film will conform to the surface of the fibers, creating a physical entanglement.

*(2) Hydrogen Bonding:* There’s a possibility for hydrogen bonding between the Nomex/Kevlar and Spirooxazine-Doped PANI. The amine groups (NH2) in PANI can form hydrogen bonds with the oxygen atoms (C=O) in the amide bonds of Nomex/Kevlar. Additionally, Spirooxazine molecules might interact with both the PANI backbone and the fibers, potentially forming additional bonding sites. These hydrogen bonds, though weak individually, can collectively contribute to a stronger interface among the components.

### 3.2. Differences between Spirooxazine-Doped PANI Thin Film and PANI Thin Film: Tools That Lead to the Selection of the Photochromic Material

*(1) Structure and Properties of the Polyaniline (PANI) Thin Film:* This section presents the chemical structure, conductivity, and color change properties.

Chemical Structure: PANI is a conducting polymer with repeating units of benzene rings connected by amine groups.

Conductivity: PANI’s electrical conductivity can be adjusted by doping with acids, making it suitable for various electronic applications.

Color Change: PANI exhibits electrochromic properties, meaning it can change color when an electrical voltage is applied. However, its response to light (photochromism) is limited.

*(2) Structure and Properties of the Spirooxazine-Doped PANI Thin Film:* This section presents the chemical structure, conductivity, and color change properties.

Chemical Structure: This composite material includes PANI doped with Spirooxazine molecules, which are photochromic compounds.

Conductivity: The incorporation of Spirooxazine may slightly affect the conductivity of PANI but generally maintains its conductive properties.

Color Change: Spirooxazine is a photochromic compound that undergoes a reversible transformation between two forms when exposed to UV light and visible light, resulting in a color change.

*(3) Electronic Structure Differences:* This section presents the electronic structure differences between the Spirooxazine-Doped PANI thin film and the PANI thin film.

PANI: The electronic structure of PANI involves *π* − *π*∗ transitions within the benzene rings and the lone pair electrons on the nitrogen atoms, which contribute to its conductivity and electrochromic properties.

Spirooxazine-Doped PANI: The presence of Spirooxazine introduces additional electronic states and transitions. Spirooxazine has a closed form and an open form, with different electronic structures. The closed form is typically colorless, while the open form, induced by UV light, is colored because of the extended conjugation system that alters its electronic absorption spectrum.

### 3.3. Advantages of Spirooxazine-Doped PANI Over PANI in Color Change: Tools That Lead to Selecting the Photochromic Material

*(1) Photochromic Response:* This section presents the photochromic response of o PANI vs. Spirooxazine-Doped PANI.

PANI: Limited photochromic properties; primarily changes color through electrochromism when voltage is applied.

Spirooxazine-Doped PANI: Exhibits strong photochromic properties due to Spirooxazine molecules. When exposed to sunlight (UV light), Spirooxazine undergoes a structural change, causing a significant color change. This process is reversible, and the material returns to its original color when the UV light is removed.

*(2) Reversibility and Speed:* This section presents the reversibility and speed of PANI vs. Spirooxazine-Doped PANI.

PANI: Electrochromic changes can be reversible but require an external voltage. The speed of the color change depends on the applied voltage and the doping level.

Spirooxazine-Doped PANI: The photochromic color change in Spirooxazine-Doped PANI is typically fast and reversible upon exposure to UV light and visible light. This allows for quick and repeatable color changes with sunlight exposure.

*(3) Sensitivity to Light:* This section presents the sensitivity to light of PANI Vs. Spirooxazine-Doped PANI.

PANI: Limited sensitivity to light, primarily responds to electrical stimuli.

Spirooxazine-Doped PANI: High sensitivity to UV light due to Spirooxazine. The color change can be triggered directly by sunlight, making it ideal for applications where visual indicators of light exposure are needed.

### 3.4. Design

*(1) Nomex Structure:* This section presents the chemical structure and atomic bonds and explains how the structure of Nomex can be represented visually.

Atomic-Level View of Nomex: Imagine long, chain-like molecules where aromatic rings (benzene rings) are linked by amide bonds (-C(=O)-NH-). The aromatic rings are arranged in a relatively stiff, rod-like structure.

Chemical Structure: Nomex (Poly(meta-phenylene isophthalamide)) is an aramid polymer consisting of meta-phenylene rings connected by isophthalamide units.

Atomic Bonds (or bonding): The atomic bonds consist of (i) phenylene rings, which are hexagonal rings made of carbon atoms, connected by alternating single and double bonds, (ii) amide groups, which are rings connected by amide linkages (-CONH-), forming hydrogen bonds with neighboring polymer chains, and (iii) hydrogen bonds, which are interactions among hydrogen atoms of the amide groups, and the oxygen atoms of other amide groups provide thermal stability and flame resistance.

Visual Representation: The Nomex structure shows meta-phenylene rings connected by isophthalamide units with hydrogen bonds among amide groups.

**Figure 1:**
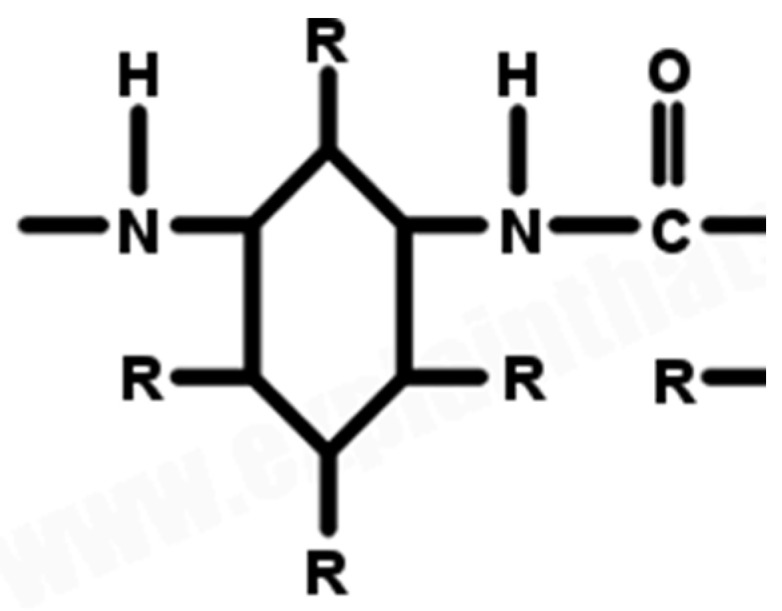
Atomic-level view of Nomex.

**Figure 2:**
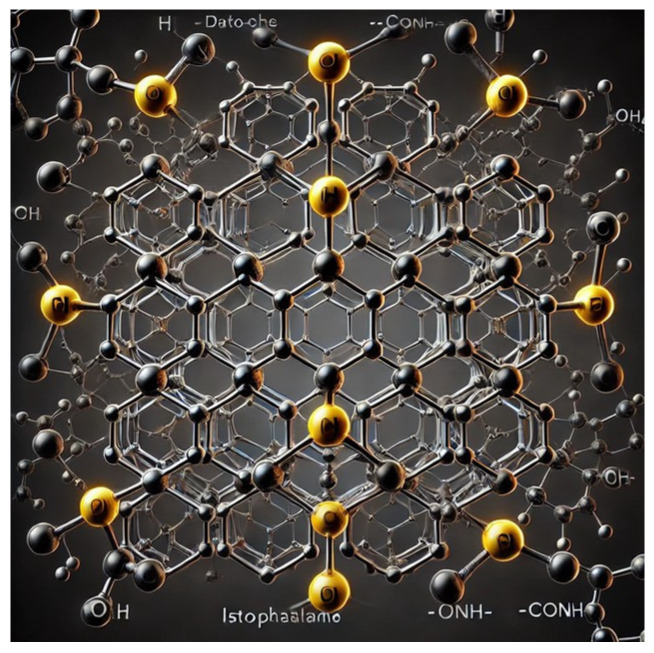
Structure of Nomex.

Figure 1 shows the atomic-level view of the designed Nomex structure, as shown in Figure 2. These figures show meta-phenylene rings connected by isophthalamide units with hydrogen bonds among amide groups. This illustration highlights the hexagonal rings made of carbon atoms, connected by alternating single and double bonds, and the amide linkages (-CONH-).

*(2) Kevlar Structure:* This section presents the chemical structure and atomic bonds and explains how the structure of Kevlar can be represented visually.

Atomic-Level View of Kevlar: Similar to Nomex, Kevlar has long chains with aromatic rings linked by amide bonds. However, the arrangement of the chains in Kevlar is more crystalline and rigid, contributing to its higher strength.

Chemical Structure: Kevlar (Poly(paraphenylene terephthalamide)) is another aramid polymer consisting of para-phenylene rings connected by terephthalamide units.

Atomic Bonds (or bonding): The atomic bonds consist of (i) phenylene rings, which are hexagonal rings made of carbon atoms, connected by alternating single and double bonds, (ii) amide groups, which are rings connected by amide linkages (-CONH-), forming extensive hydrogen bonds among polymer chains, and (iii) hydrogen bonds, which are strong interactions among hydrogen atoms of amide the groups, and the oxygen atoms of other amide groups contribute to Kevlar’s high tensile strength and stiffness.

Visual Representation: The structure of Kevlar shows para-phenylene rings connected by terephthalamide units with an extensive hydrogen bonding network.

**Figure 3:**
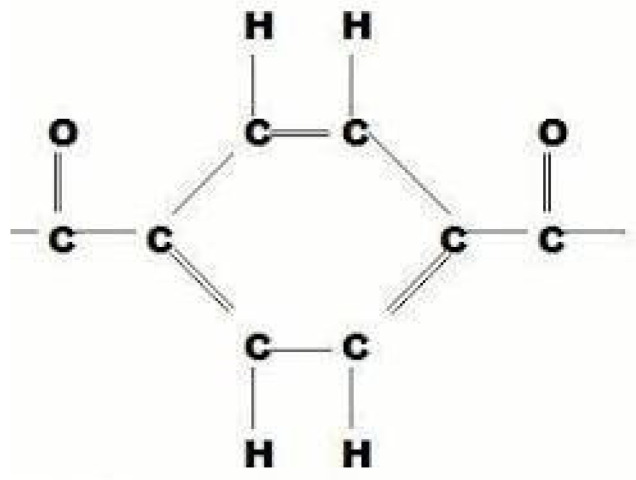
Atomic-level view of Kevlar.

**Figure 4:**
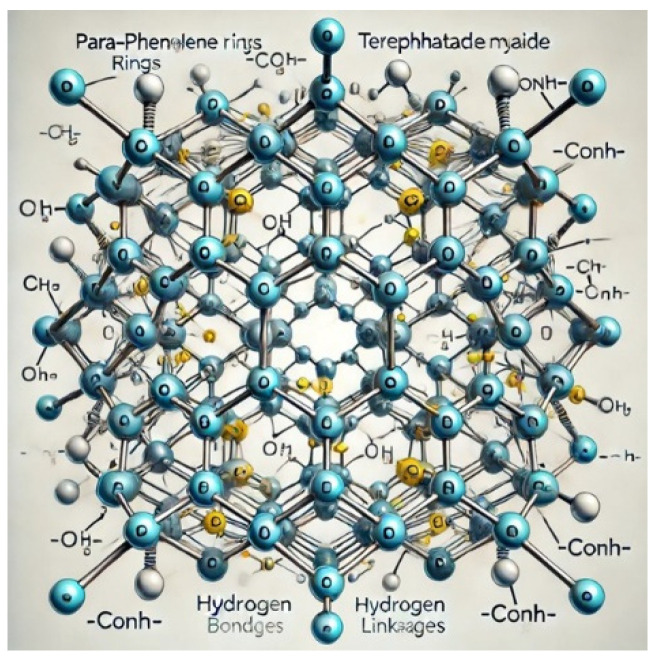
The structure of Kevlar.

Figure 3 shows the atomic-level view of the designed Kevlar structure, as shown in Figure 4. These figures show para-phenylene rings connected by terephthalamide units with an extensive hydrogen bonding network. This illustration highlights the hexagonal rings made of carbon atoms, connected by alternating single and double bonds, and the amide linkages (-CONH-).

*(3) Spirooxazine-Doped PANI Thin Film:* This section presents the chemical structure and the atomic bonds and explains how Spirooxazine-Doped PANI can be represented visually.

Atomic-Level View of the Polyaniline (PANI) thin film:

Imagine a network of interconnected aniline rings (benzene rings with an attached amine group -NH2). The bonding among these rings involves alternating single and double bonds, allowing for the movement of electrons along the chain and contributing to conductivity.

Atomic-Level View of Spirooxazine-Doped PANI: Imagine a network of interconnected aniline rings (benzene rings with an attached amine group -NH2). The bonding among these rings involves alternating single and double bonds, allowing for the movement of electrons along the chain and contributing to conductivity. Incorporated within this network are Spirooxazine molecules, which are photochromic compounds. These Spirooxazine molecules are bonded to the Polyaniline chains, potentially through interactions with the amine groups or the aromatic rings, creating points where the Spirooxazine can influence the electronic properties of the PANI, such as by altering its conductivity or optical properties in response to light.

Below is the atomic-level view of Spirooxazine-Doped Polyaniline (PANI), showing a network of interconnected aniline rings with alternating single and double bonds, along with incorporated Spirooxazine molecules bonded to Polyaniline chains. This illustration highlights the interactions between Spirooxazine and PANI, influencing its electronic and optical properties. The second image shows the Polyaniline backbone with incorporated Spirooxazine molecules and highlights the interaction points where Spirooxazine molecules are bonded to the Polyaniline chain

Chemical Structure: PANI (Polyaniline) is a conducting polymer with repeating units of benzene rings (C6H4) connected by amine (NH) groups. Spirooxazine molecules are incorporated into the PANI matrix. • Atomic Bonds (or bonding): The atomic bonds consist of (i) benzene rings, which are hexagonal rings made of carbon atoms, connected by alternating single and double bonds, (ii) amine groups, which are benzene rings connected by amine groups, allowing for interactions with other materials, and (iii) Spirooxazine interactions, in which Spirooxazine molecules interact with PANI chains, potentially through the amine groups or aromatic rings, modifying the conductivity and optical properties of PANI.

**Figure 5:**
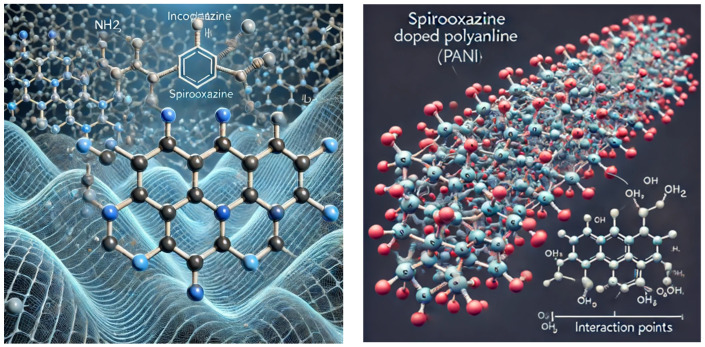
Atomic-level view of Spirooxazine-Doped PANI.

Visual Representation: The figures provided above (Figure 5) show the designed Spirooxazine-Doped PANI structure with benzene rings connected by amine groups and Spirooxazine molecules interacting with the PANI matrix.

*(4) Composite Material Made Up of Nomex, Kevlar, and Spirooxazine-Doped PANI:* This section presents the design concept and bonding and how the composite material can be represented visually.

Design Concept: The composite is designed with interwoven or layered Nomex and Kevlar polymer chains. The Spirooxazine-Doped PANI thin film is deposited onto the network composite material made up of Nomex and Kevlar.

Bonding and Interactions: The bonding consists of (i) hydrogen bonds between the amide groups of Nomex and Kevlar, as well as between these groups and the Spirooxazine-Doped PANI thin film, and (ii) Van der Waals interactions, which are additional interactions between the Spirooxazine-Doped PANI film and the Nomex/Kevlar network that enhance the composite’s properties.

**Figure 6:**
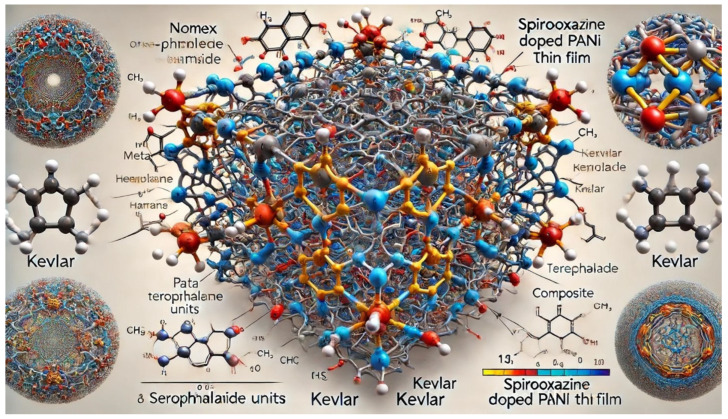
Design of the composite material made up of Nomex, Kevlar, and Spirooxazine-Doped Polyaniline (PANI) thin film.

Visual Representation: The composite structure in Figure 6 shows interwoven Nomex and Kevlar units with a Spirooxazine-Doped PANI thin film deposited onto the network, illustrating hydrogen bonds and other interactions among the materials.

Figure 6 shows the atomic-level view of the composite structure made up of Nomex, Kevlar, and the Spirooxazine-Doped Polyaniline (PANI) thin film. This illustration shows Nomex and Kevlar fibers interwoven to form a strong, three-dimensional network with the Spirooxazine-Doped PANI thin film deposited onto the network. It highlights the physical entanglement and potential hydrogen bonding between the amine groups (NH2) in PANI and the oxygen atoms (C=O) in the amide bonds of Nomex/Kevlar, along with the interaction between Spirooxazine molecules and both the PANI backbone and the fibers. The image highlights the hydrogen bonding and structural features of each material, as well as their interactions in the composite structure.

## 4. Equations and Parameters Considered

### 4.1. Equations and Parameters for the Mechanical Investigation

The parameters employed in the mechanical analysis of Nomex and Kevlar, along with the PANI film thickness of 0.02 m, are detailed in our previously published article (refer to [8]). The thickness of the Spirooxazine-Doped PANI matches that of the PANI thin film, as shown in our previously published article (refer to [8]). Additionally, for a thorough understanding of the azimuthal mode numbers, load magnitudes, and the equations utilized in the mechanical investigation, we encourage referencing the same published article (refer to [8]). This publication serves as an invaluable resource, presenting detailed methodologies and findings to support enhanced comprehension and future research endeavors.

### 4.2. Equations and Parameters for the Thermal Investigation

In our previously published article (refer to [8]), detailed parameters regarding our Nomex/Kevlar/PANI film material are provided, offering specific insights into (i) material radius, (ii) material thickness, (iii) rotational speed, (iv) laser movement time, (v) laser beam radius, (vi) material surface emissivity, and (vii) laser power. Moreover, all equations employed in our study, encompassing heat transfer, thermal insulation, and surface-to-ambient radiation, are drawn from the same publication (refer to [8]). The equations and parameters considered for the Spirooxazine-Doped PANI are similar to the parameters and equations considered for the PANI thin film as shown in our previously published article (refer to [8]).

## 5. Simulation and Results

### 5.1. Results for the Mechanical Investigation of the Composite Material Designed at the Polymer Level

The layerwise theory uses three-dimensional kinematics and can predict stresses and strains with high accuracy. The results obtained using this theory are therefore used as a benchmark. The results obtained using the ESL theory and the multiple-model method are compared with the layerwise theory predictions.

The layerwise approach employs three-dimensional kinematics, providing precise forecasts of stresses and strains [8]. These outcomes are consequently adopted as a standard of comparison. The outcomes derived from the ESL theory [8] and the multiple-model approach [8] are juxtaposed against the layerwise theory forecasts [8]. Note that the identical methodologies and parameters utilized in this section of this study were consistently applied in our prior publication [8].

**Figure 7:**
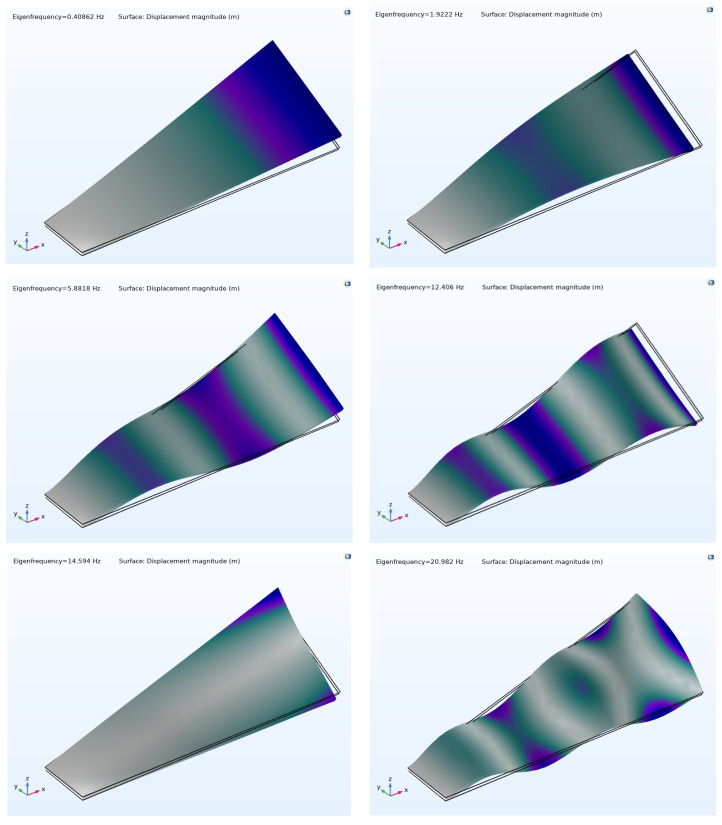
The first six mode shapes and corresponding eigenfrequencies of the composite material, using the multiple-model method.

*(1) Eigenfrequency Analysis:* The initial six eigenmodes, computed using the multiple model method, are illustrated in Figure 7. Eigenmodes obtained through the layerwise and ESL theories closely resemble those derived from the multiple-model method and are therefore not included here.

However, discrepancies in the calculated eigenfrequencies are evident across the different modeling techniques. Table 3 presents the corresponding six eigenfrequencies for each method. It is important to note that the same approach employed in this section of this study was consistently utilized in our previous publication [8].

Analysis of the data presented in the provided table highlights a striking concurrence between the cost-effective projections generated by the multiple-model method and those of the layerwise approach. Discrepancies emerge in forecasts utilizing the ESL theory, likely stemming from its inherent computational efficiency, albeit with reduced accuracy for moderately thick shells. This underscores the computational advantage of adopting a multiple-model approach, wherein thicker segments of a composite structure are simulated using the layerwise theory, while the ESL theory is applied to thinner sections.

**Figure 8:**
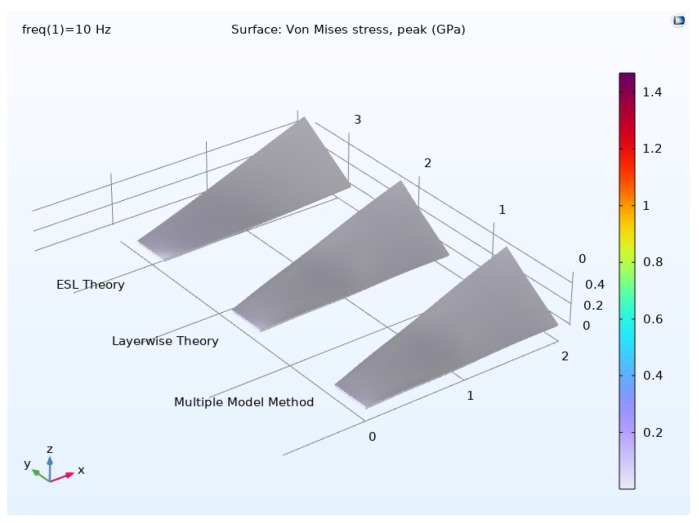
Peak von Mises stress distribution in the composite material.

*(2) Frequency Domain Analysis:* Figure 8 showcases the graphical representation of the von Mises peak stress distribution for each modeling technique. The illustration demonstrates a notable similarity between the stress patterns produced by the multiple-model method and the layerwise approach, both in their distribution and peak intensity. In contrast, a slight variation is observed in the stress distribution when utilizing the ESL theory.

**Figure 9:**
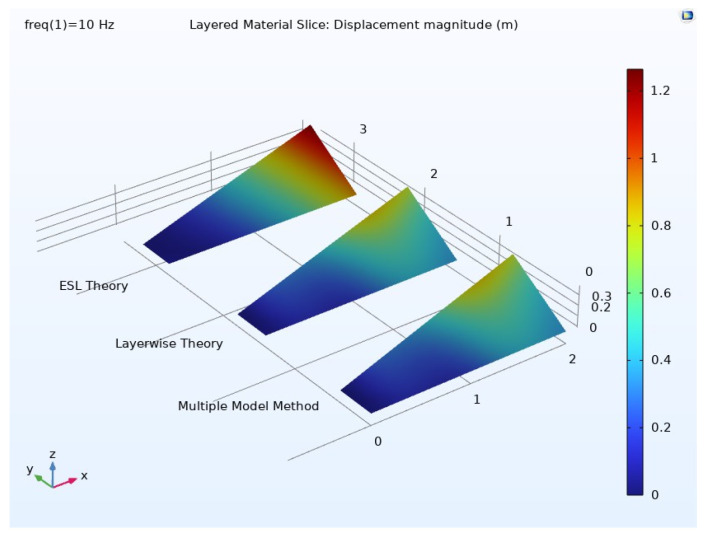
Displacement in the top layer of the composite material.

The frequency domain results regarding displacements are presented in Figure 9 for each modeling approach. Once again, the outcomes obtained from the multiple-model method closely match those of the layerwise theory, whereas the ESL theory shows inaccuracies in predicting both displacement distribution and peak values.

This section delves into the examination of the peak von Mises stress distribution across the thickness direction at a specific point.

The results obtained from the multiple-model method and the layerwise theory show significant similarity, in contrast to the ESL theory’s divergent results. This variation is expected, considering the ESL theory’s limitations in accurately computing inter-laminar shear stresses in thick composites.

### 5.2. Results for the Thermal Investigation of the Composite Material Designed at the Polymer Level

**Figure 10:**
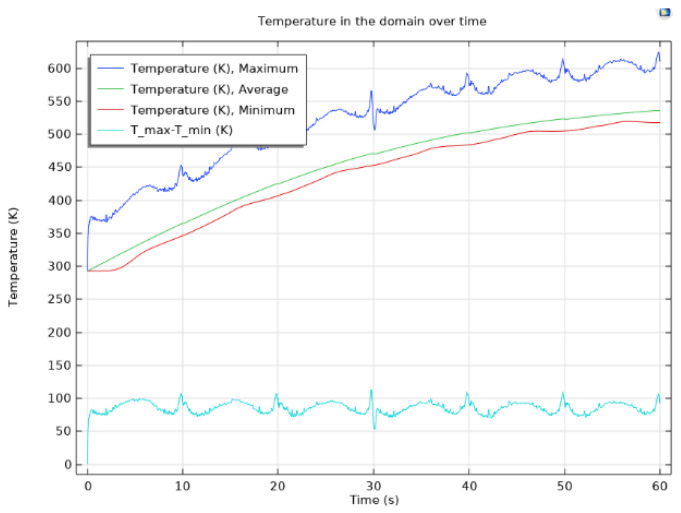
Maximum, minimum, and average temperatures of the composite material as functions of time.

**Figure 11:**
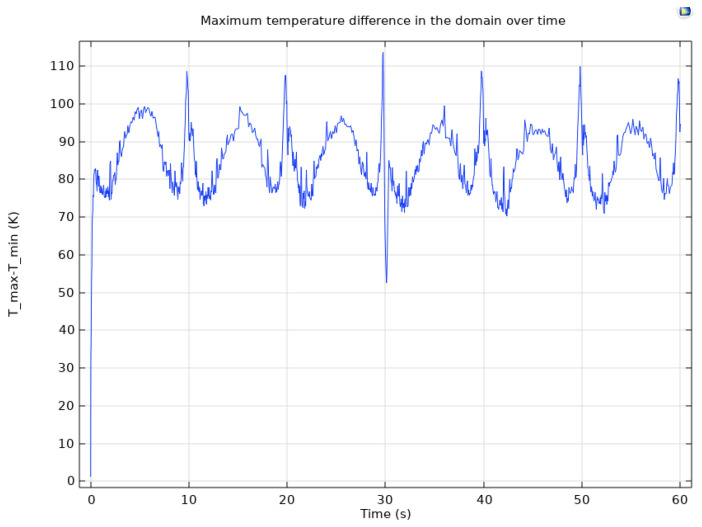
Difference between the maximum and minimum temperatures on the composite material.

In Figure 10, probe plots display the maximum, minimum, and average temperatures of the designed composite material, offering valuable insights into its thermal behavior. Figure 11 further enhances our understanding by showcasing the temperature difference between the maximum and minimum temperatures, elucidating temperature differentials across the material.

Figure 10 provides probe representations indicating the highest, lowest, and mean temperatures of the engineered composite material, allowing for a comprehensive analysis of thermal characteristics. Furthermore, Figure 11 offers a detailed probe depiction highlighting the temperature variance between the highest and lowest points, emphasizing areas of temperature disparity within the material.

**Figure 12:**
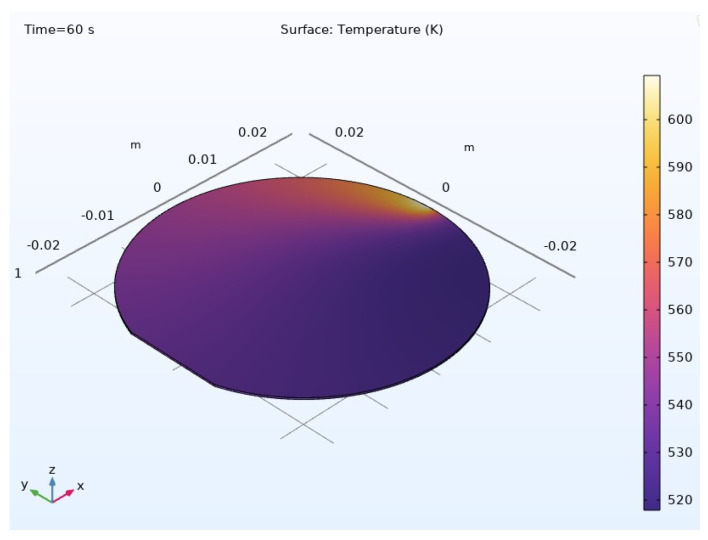
Temperature variation across the composite material.

**Figure 13:**
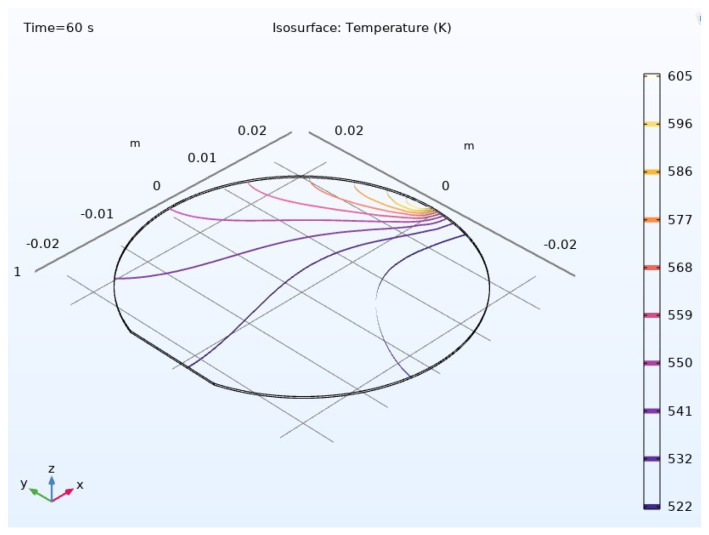
Isosurface temperature across the composite material.

The temperature distribution throughout the composite material is visually represented in Figure 12 and Figure 13, providing a holistic view of thermal gradients and variations across its surface. These graphical representations offer valuable insights into the thermal performance and behavior of the composite material, aiding in its optimization and further development for various applications.

The thermal profile exhibits significant temperature fluctuations as the laser uniformly distributes heat across a broader swept region when directed towards the periphery of the composite material. This observation underscores the dynamic nature of heat distribution and highlights the importance of understanding thermal behavior for optimizing the material’s performance in various applications.

### 5.3. Results and Discussion of the Experimental Investigation of the Composite Material Designed at the Polymer Level

As part of the experimental investigation, a PANI thin film, representing a small section of the composite material, was manufactured. We followed the methodology outlined in the previous section of this paper, ensuring consistency and precision in this study.

*(1) Morphology:* The outcomes and discussion stemming from our experimental exploration are available for reference in our previously published article (refer to [10]). These comprehensive results and discussions delve into the material’s morphology, as depicted by the SEM images of the PANI film we obtained. We encourage a thorough examination of that article to gain deeper insights into our findings and analyses.

The findings indicate that the PANI film exhibits appealing traits, positioning it as a promising contender for integration into composite materials for optical mining ventures in space. Notably, SEM imaging unveils a porous, seaweed-like structure in the electrodeposited PANI films. This morphology, characterized by suspended rods and porosity, hints at conducive circumstances for efficient ion conveyance. The porous film suggests heightened mechanical resilience owing to the amplified surface area, a boon for enduring the rigors of space, including potential micrometeoroid impacts and temperature fluctuations.

*(2) Colorimetric Properties from CIE Studies:* The findings and discourse on the colorimetric properties are detailed in our previously published article (refer to [10]). Within that publication, in-depth analyses and interpretations that shed light on the intricate aspects of these properties are presented.

The findings suggest the PANI film exhibits favorable traits, rendering it a viable option for inclusion in composite materials for optical mining in space. The analysis of the colorimetric data and images from our previously published article [10] demonstrates PANI’s exceptional linearity and reversibility during coloration and bleaching. The fluctuation in chromaticity values reflects the film’s rapid and reversible color transitions, vital for responsive optical properties in mining operations.

*(3) Optical Modulation:* For a thorough examination of the colorimetric properties, it is important to refer to our previously published article (refer to [10]). Within its pages, detailed results and discussions that delve into the nuances of these properties are presented. Additionally, this article provides valuable insights and implications for further research in this field.

The findings from our previously published article indicate that the PANI film possesses advantageous traits, rendering it a promising candidate for integration into composite materials for space-based optical mining endeavors. Optical modulation experiments underscore PANI’s capacity for distinct color variations in reaction to diverse electrical inputs. The film exhibits a spectrum of hues, swiftly responding to voltage changes with shifts from light green to green and blue tones, as seen in our published article (see [10]). This dynamic transition showcases the film’s adaptability and responsiveness, crucial for real-time control in optical mining operations.

*(4) Raman Spectroscopy:* In-depth exploration of the data obtained in our Raman spectroscopy studies is presented in our previously published article (refer to [10]). Within its pages, comprehensive results and discussions that illuminate our findings are presented. Additionally, this article serves as a valuable resource for understanding the implications of our research and its potential applications.

The findings show that PANI films exhibit favorable traits, rendering them a viable option for integration into space-grade composite materials for optical mining. The distinctive phases identified in PANI film Raman spectra, denoting diverse molecular configurations and oxidation levels, signify the material’s capacity for structural adjustments across varied circumstances. The transition from benzene to quinone phases highlights the film’s resilience to oxidation [10], crucial for enduring extreme space temperatures.

*(5) Cyclic Voltammetry in EC Films:* A comprehensive understanding of the data derived from Cyclic Voltammetry in EC film studies is presented in our previously published article (see [10]). This publication offers a thorough overview of the results and discussions stemming from our research, providing valuable insights into the electrochemical behavior of the films. It also highlights implications for further investigation and potential applications in this domain.

Our previously published article (see [10]) highlights the favorable properties exhibited by the PANI film, positioning it as a promising candidate for integration into composite materials for in-space optical mining applications. Specifically, the cyclic voltammogram of the PANI film reveals the electrochemical processes underlying color modulation, showcasing the material’s responsiveness to external stimuli such as voltage. The transitions between different oxidation states and corresponding color changes emphasize the film’s versatility and potential for use in optical mining applications, where precise control over material properties is crucial.

### 5.4. Simulation Results for the Composite Material Modeled and Designed at the Atomic and Molecular Levels

**Figure 14:**
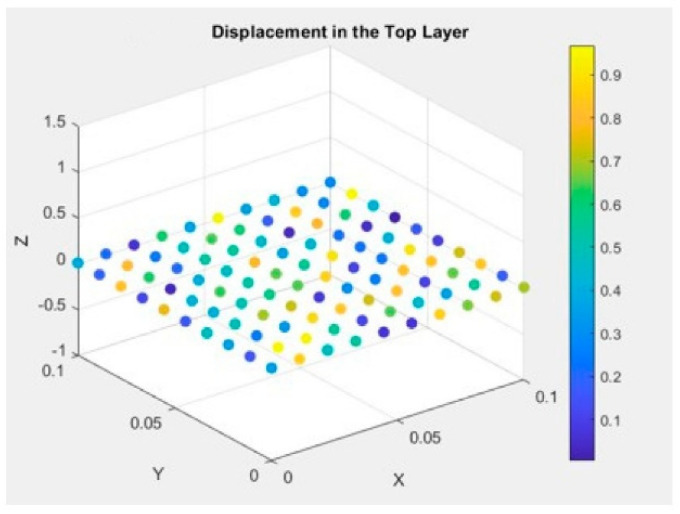
Displacement of the composite material made up of Nomex, Kevlar, and Spirooxazine-Doped Polyaniline (PANI) thin film.

*(1) Results for the Mechanical Investigation:* Figure 14 illustrates the von Mises peak stress distribution in a graphical format. When comparing these results to those obtained from materials designed and modeled at the polymer level, it is evident that the composite material, comprising Nomex, Kevlar, and Spirooxazine-Doped PANI, designed and modeled at the atomic and molecular levels, exhibits superior high-strength properties for use in asteroid mining.

**Figure 15:**
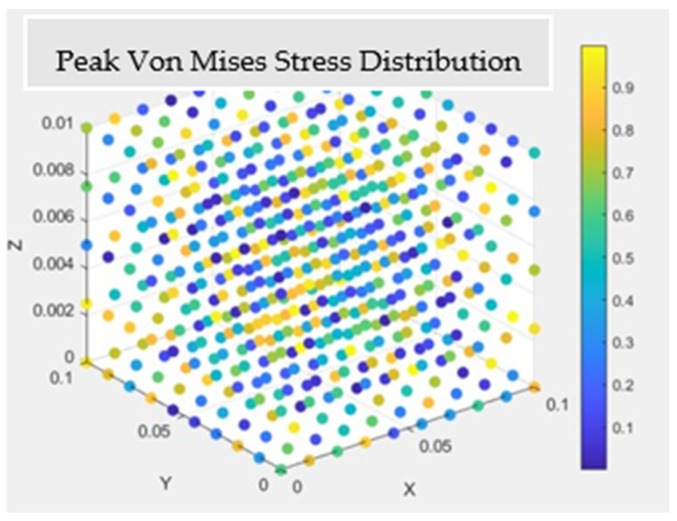
Peak von Mises stress distribution in the composite material made up of Nomex, Kevlar, and Spirooxazine-Doped Polyaniline (PANI) thin film.

Figure 15 presents the frequency domain results concerning displacements. Again, these results demonstrate that the composite material, engineered at the atomic and molecular levels, surpasses the performance of materials designed and modeled at the polymer level, making it an excellent choice for high-strength applications in asteroid mining.

**Figure 16:**
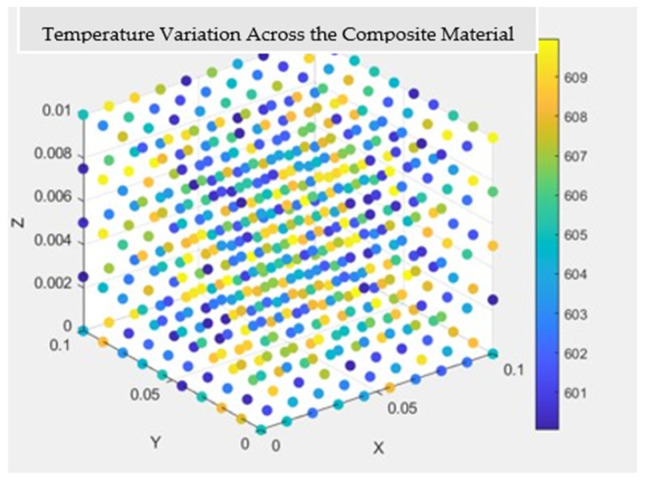
Temperature variation across the composite material made up of Nomex, Kevlar, and Spirooxazine-Doped Polyaniline (PANI) thin film.

*(2) Results for the Thermal Investigation:* Figure 16 visually depicts the temperature distribution across the composite material, offering a comprehensive view of the thermal gradients and variations within the Nomex, Kevlar, and Spirooxazine-Doped Polyaniline (PANI) thin film. When compared to results from materials designed and modeled at the polymer level, the composite material, engineered at the atomic and molecular levels, demonstrates superior performance as a high-strength material for asteroid mining.

## 6. Conclusions

This study simulated the mechanical and thermal behaviors of a composite material consisting of Nomex, Kevlar, and Spirooxazine-Doped PANI film at both the polymer and atomic/molecular levels. Experimental testing was performed in a laboratory on a sample manufactured from the composite material designed and modeled at the polymer level.

Initially, this study illustrated the modeling of a composite structure composed of Nomex, Kevlar, and Spirooxazine-Doped PANI film. Three modeling techniques, layerwise theory, equivalent single layer (ESL) theory, and a multiple-model method, were employed. Modal and frequency–response analyses were conducted, comparing the multiple-model method with traditional layerwise and ESL theories in terms of performance and accuracy, including the through-thickness stress distribution. The results indicate the multiple-model method is the optimal choice for accuracy and performance in modeling composite structures.

The combination of Nomex, Kevlar, and Spirooxazine-Doped PANI film is identified as optimal for in-space optical mining applications.

Secondly, the composite material made up of Nomex, Kevlar, and Spirooxazine-Doped PANI film was modeled and simulated to determine its mechanical strength and response to laser heating as performed similarly in our previously published article. The transient thermal response of the material to radial laser heating was obtained, with peak, average, and minimum temperatures computed. Thermodynamics simulations indicate suitability for high-temperature resistant composite material in-space optical mining applications.

Thirdly, experimental investigations on the electrodeposited PANI film’s colorimetric and electrochromic characteristics were conducted [10]. Cyclic voltammetry tests reveal ion-diffusion dominant processes in electrochromism [10]. Significant transitions between benzene and quinone phases are observed in Raman spectra of PANI [10]. Infrared camera testing demonstrates the PANI film’s capacity to block infrared radiation, indicating potential applications in satellite thermal management and military camouflage [10].

Overall, the PANI film exhibits desirable properties for incorporation into composite materials for in-space optical mining applications, promising mechanical strength, thermal stability, and optical responsiveness [10].

In summary, our simulations suggest the composite material made up of Nomex, Kevlar, and Spirooxazine-Doped PANI film material is suitable for high-strength, high-temperature resistant composite material for in-space optical mining applications.

## Figures and Tables

**Table 1:** Material properties.

Material Properties	Values
Poisson ration (*v*)	0.35
Young’s modulus (*E*)	500 GPa
Shear modulus (*G*)	1 × 10^6^ N/m^2^
Density (Kg/m^3^)	1200 Kg/m^3^

**Table 2:** Material thickness and corresponding mesh elements.

Material	Thickness (mm)	Mesh Elements
Nomex	1.0	8
Kevlar	1.0	16
PANI film	0.75	10

**Table 3:** Corresponding six eigenfrequencies for each method.

Multiple Model (Hz)	Layerwise (Hz)	ESL (Hz)
0.40862	0.40925	6.0973
1.9222	1.9281	23.614
5.8818	5.9016	33.842
12.406	12.456	72.964
14.594	14.606	90.215
20.982	21.078	132.34

## Data Availability

The raw data supporting the conclusions of this article will be made available by the authors on request.
